# Aerobic Exercise Training Inhibits Neointimal Formation via Reduction of PCSK9 and LOX-1 in Atherosclerosis

**DOI:** 10.3390/biomedicines8040092

**Published:** 2020-04-19

**Authors:** Wei Li, Heegeun Park, Erling Guo, Wooyeon Jo, Kyu Min Sim, Sang Ki Lee

**Affiliations:** 1 Department of Sport Science, College of Natural Science, Chungnam National University, 99 Daehak-ro, Yuseong-gu, Daejeon 34134, Korea; ty1986@zjnu.edu.cn (W.L.); exepre@cnu.ac.kr (H.P.); chuang042@gmail.com (E.G.); dndus7942@naver.com (W.J.); skm477@naver.com (K.M.S.); 2 Exercise and Metabolism Research Center, College of Physical Education and Health Sciences, Zhejiang Normal University, Jinhua 321004, China

**Keywords:** atherosclerosis, aerobic exercise, endothelial dysfunction, PCSK9, LOX-1

## Abstract

The purpose of this study was to investigate whether aerobic exercise training inhibits atherosclerosis via the reduction of proprotein convertase subtilisin/kexin type 9 (PCSK9) expression in balloon-induced common carotid arteries of a high-fat-diet rats. Male SD (Sprague Dawley) rats fed an eight-weeks high-fat diet were randomly divided into three groups; these were the sham-operated control (SC), the balloon-induced control (BIC) and the balloon-induced exercise (BIE). The aerobic exercise training groups were performed on a treadmill. The major findings were as follows: first, body weight gain was significantly decreased by aerobic exercise training compared to the BIC without change of energy intake. Second, neointimal formation was significantly inhibited by aerobic exercise training in the balloon-induced common carotid arteries of high-fat-diet rats compared to the BIC. Third, low-density lipoprotein (LDL) receptor (LDLr) expression was significantly increased by aerobic exercise training in the livers of the high-fat diet group compared to the BIC, but not the proprotein convertase subtilisin/kexin type 9 (PCSK9) expression. Fourth, aerobic exercise training significantly decreased the expression of PCSK9, the lectin-like oxidized LDL receptor-1 (LOX-1), and vascular cell adhesion molecule-1 (VCAM-1) in balloon-induced common carotid arteries of high-fat-diet rats compared to the BIC. In conclusion, our results suggest that aerobic exercise training increases LDLr in the liver and inhibits neointimal formation via the reduction of PCSK9 and LOX-1 in balloon-induced common carotid arteries of high-fat-diet-induced rats.

## 1. Introduction

Atherosclerosis is a condition in which an artery is occluded by a plaque that leads to cardiovascular disease, with high morbidity and mortality [1]. A high-fat diet (HFD) is the main factor that causes atherosclerosis, such as elevated serum levels of low-density lipoprotein cholesterol (LDL-C) and oxidative stress thereby promoting endothelial damage [2,3].

Previous studies have indicated that lectin-like oxidized LDL (low-density lipoprotein) receptor-1 (LOX-1) binds to oxidized LDL (ox-LDL) in vasculature, which plays an important role in endothelial damage [4]. High levels of LDL-C, particularly in the form of ox-LDL, were shown to increase intracellular reactive oxygen stress (ROS) generation via LOX-1 activation [5]. Numerous studies have shown that the reduction of the LOX-1 expression can significantly delay the development of atherosclerosis [6,7].

Proprotein convertase subtilisin/kexin type 9 (PCSK9) was initially proposed in 2003 [8]. It is mainly produced and secreted in the liver and is expressed in the small intestine, kidneys and brain [9,10]. PCSK9 overexpression increases intracellular LDL receptor (LDLr) degradation in hepatic cells [11] and the circulation of PCSK9 inhibits LDLr recycling to the cell surface [12,13]. By contrast, the inhibition of PCSK9 significantly reduces plasma LDL-C concentration [14], thus preventing lipid accumulation on the vessel wall and inhibiting atherogenic plaque formation [15]. Interestingly, Ferri et al. demonstrated that PCSK9 was detectable in human atherosclerotic plaques and PCSK9 activation reduced LDLr expression in macrophages [16]. These results indicated that PCSK9 could directly affect the formation of foam cells and atherosclerosis.

Current understanding suggests that exercising regularly as a therapeutic strategy can effectively inhibit atherosclerosis [17,18]. Exercise training improves damaged endothelial cells (ECs) and inhibits neointimal formation, which prompts the occurrence of atherosclerosis [19]. Exercising regularly can also markedly reduce plasma LDL-C and ox-LDL during oxidative stress [20,21]. It markedly decreases LOX-1 expression and mediates ox-LDL-induced apoptosis in vascular tissues [22]. However, studies on the effect of exercise on the prevention of atherosclerosis via the LOX-1-mediated pathway in the molecular aspect are rarely conducted.

Several studies have indicated that exercise could also reduce plasma PCSK9 concentration in high-fat-diet-induced mice [23] and decrease hepatic PCSK9 mRNA in ovariectomized rats [24]. This study mainly focused on the role of exercise on PCSK9 in the liver [24,25] and the intestine [26] or the application of drugs, such as antibodies, to PCSK9 to reduce the risk of atherosclerosis [27,28]. However, it did not establish the effect of exercise training on the relationships of PCSK9 and LDLr in the livers and PCSK9 and LOX-1 in atherosclerotic regions of high-fat-diet rats.

Therefore, this study was conducted to determine whether aerobic exercise training contributes to treat atherosclerosis via PCSK9 and LOX-1 in balloon-induced common carotid arteries of high-fat-diet rats.

## 2. Materials and Methods

### 2.1. Experimental Animals

Six-week-old male Sprague-Dawley (SD, *n* = 30) rats were used in this experiment. The rats were adapted to a new environment for one week, and then fed a 60% high-fat (D12492, Open Source Diets, Research Diets, New Brunswick, NJ, USA) and a chow-diet for 8 weeks [23]. After 8 weeks, they were randomly divided into SC (sham-operated control group, *n* = 10), BIC (balloon-injured control group, *n* = 10), and BIE (balloon-injured exercise group, *n* = 10), respectively. The rats were kept in animal cages (30 cm × 20 cm) by respective groups (temperature 20–25 °C, humidity 50–60% and contrast 12-h cycle). Diet-induced obesity was created by feeding a high-fat diet (60% fat, Raon Bio, Korea) ad libitum until the end of experiment. The food intake was measured every week. All experiments were approved by the Animal Care and Use Committee at the Chungnam National University (CNU-00818, 4 October 2016).

### 2.2. Balloon Injury Model

A balloon induced-atherosclerosis rat model was used in this experiment [29]. An anesthetic (a mixture of 80 mg/kg ketamine and 12 mg/kg xylazine) was injected intraperitoneally and then a 2 F Forgaty Catheter (Edwards Lifesciences, Irvine, CA, USA) was inserted into the left common carotid artery (CCA) through an external carotid artery (ECA). A pressure gauge was used to inflate the catheter balloon 1.5 times greater than the diameter of the carotid artery and then a 10 mm injury was induced by the withdrawal of inflated balloon catheter 5 times. The rats were allowed at least 3 days to recover from the surgery.

### 2.3. Exercise Protocol

The rats were exercised on a treadmill (motorized rodent treadmill) for 8 weeks in order to investigate the effects of aerobic exercise. The rats ran at 10 m/min with a 0% incline for 10 min on the first day. The speed and duration of the exercise were increased by 10 min and 2 m/min every day until the fourth day. From the fifth day to the end of the experiment, the rats ran at 16 m/min for 60 min [17]. This exercise intensity corresponded to 65–70% of the maximal oxygen uptake.

### 2.4. Experimental Animals

The rats were maintained on a feeding program and their individual body weights were recorded every week throughout the experimental period. In order to measure the food intakes, 120 g of diets per cage (3 rats) were supplied and the remaining amounts of supplied diets were measured two times per week after using an automatic electric balance (Cas, Seoul, Korea). These were regarded as the individual mean daily food consumption of rats (g/day/rats).

### 2.5. Hematoxylin and Eosin (H & E) Staining

In order to measure the neointimal formation, the balloon-injured rat carotid arteries were fixed in 4% formaldehyde and were paraffin-embedded. Serial cross sections (5 μm thick) of arteries were stained with hematoxylin and eosin (MHS-32, Sigma-Aldrich, MO, USA). A DP70 camera (Olympus, Tokyo) and a TSView version 7 (Fuzhou Tucsen Image Technology, Japan) were used to measure the size (μm^2^) of the intima, media and lumen, to calculate the intima-media thickness and lumen diameter so as to compare the degree of the neointimal formation.

### 2.6. Western Blotting

The tissues of the carotid arteries and liver were homogenized in lysis buffer (20 mM Tris-HCl, 0.5% NP-40, 250 mM NaCl, 3 mM EDTA, 3 mM EGTA, 2 mM DTT, 0.5 mM phenylmethylsulfonylfluoride, 2 mM b-glycerophosphate, 1 mM sodium orthovanadate, 1 ug/mL leupeptin and pH 7.5, Sigma, USA), and then centrifuged for 30 min at 14,000 rpm at 4 °C so as to remove the supernatant and quantify the proteins by using a BCA assay kit (Bio-rad, Rockford, IL, USA). In addition, 50 ug of proteins were subjected to electrophoresis on a 9% SDS-PAGE and then transferred to a polyvinylidene difluoride (PVDF) membrane. The nonspecific reaction of the membrane was removed by blocking it for one h at room temperature in 5% nonfat dry milk in Tris buffered saline Tween-20 (TBST). PCSK9 (abcam, San Francisco, CA, USA), LDLr (Abcam, San Francisco, CA, USA), LOX-1 (Abcam, San Francisco, CA, USA), VCAM-1 (Cell signaling, USA) and beta-actin (Sigma, St. Louis, MO, USA) were incubated for 18 h at 4 °C in TBST with 5% nonfat dry milk. Horseradish peroxidase (HRP)-conjugated rabbit-antimouse IgG (Calbiochem, San Diego, CA, USA) was used for secondary antibodies. Blots were developed for visualization by using an enhanced chemiluminescence detection kit (Pierce Biotechnology, Rockford, IL, USA), and the Image Quant software (Molecular dynamics, Sunnyvale, CA, USA) was used to quantify the expression.

### 2.7. Statistical Analysis

SPSS 24.0 statistics were used to calculate the descriptive statistics quantity from the results of this study and a one-way ANOVA was used to verify each variable, while Duncan’s test was used for post-hoc analysis. The differences were considered statistically significant at *p* < 0.05.

## 3. Results

### 3.1. Aerobic Exercise Training Inhibited Body Weight Gain

**Figure 1:**
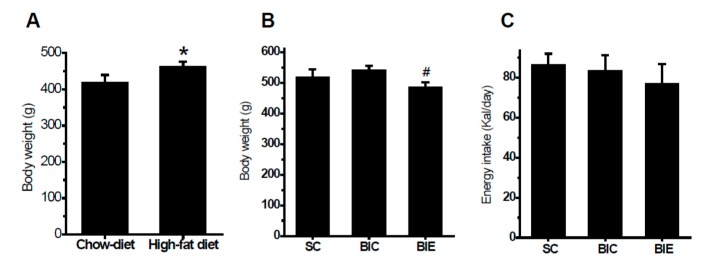
Aerobic exercise training significantly attenuated body weight without a change of energy intake in the balloon-induced rats with a high-fat diet. Body weight after 8 weeks of a high-fat diet (**A**), body weight after 8 weeks of exercise (**B**), and energy intake (**C**). Chow-diet (*n* = 10), high-fat diet (*n* = 30), SC (*n* = 10), sham control; BIC (*n* = 10), balloon-injured control; BIE (*n* = 10), balloon-injured exercise. Data showed a mean ± S.E. * *p* < 0.05 vs. Chow-diet; ^#^ *p* < 0.05 vs. BIC.

After the high-fat-diet induced weight gain was compared with the chow-diet groups for eight weeks (*p* < 0.05, 420.1 ± 19.2 vs. 463.9 ± 12.5) (Figure 1A), we investigated whether eight weeks of aerobic exercise training reduced the final body weight in the balloon-induced atherosclerotic rat model with a high-fat diet (Figure 1B,C). The high-fat diet significantly increased body weight gain compared with the chow-diet groups. Aerobic exercise training significantly decreased body weight gain compared with the SC and BIC groups (*p* < 0.05, 520 ± 24.4 and 543.9 ± 11.8 vs. 486.7 ± 14.3) without a change of energy intake (*p* > 0.05).

### 3.2. Aerobic Exercise Inhibited Neointimal Formation

**Figure 2:**
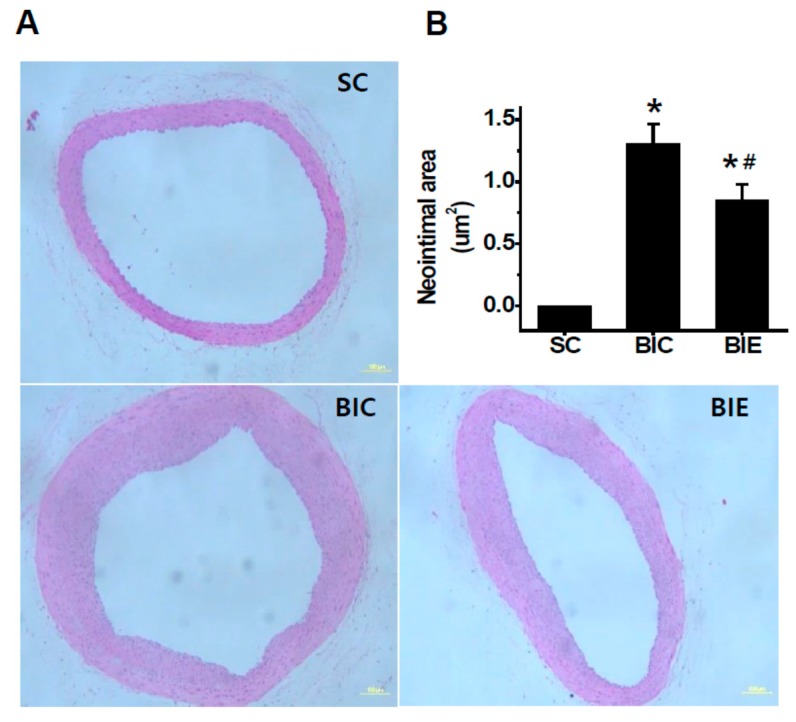
Aerobic exercise training inhibited the neointimal formation in the balloon-injured common carotid arteries (CCA) of the balloon-induced rats with high-fat diets. Representation of hematoxylin and eosin (H&E) staining in the injured CCA (**A**). Neointima of the cross-sectional area (μm^2^) was quantified by planimetry (**B**). SC (*n* = 7), sham Control; BIC (*n* = 8), balloon-injured control; BIE (*n* = 8), balloon-injured exercise. Data showed a mean ±S.E. * *p* < 0.05 vs. SC; ^#^ *p* < 0.05 vs. BIC. Microscope were used by 100X and Scale bars are 100 μm.

In order to investigate the effect of aerobic exercise on the neointimal formation in the balloon-induced rat model with a high-fat diet, we measured the neointimal formation in the balloon-injured common carotid arteries (CCA) with a high-fat diet (Figure 2). The neointimal formation was significantly inhibited by the aerobic exercise training compared to the BIC group (*p* < 0.05, 1.31 ± 0.15 vs. 0.85 ± 0.12).

### 3.3. Aerobic Exercise Increased LDLr Expression in Liver of Rats, but Did not Affect PCSK9 Expression

**Figure 3:**
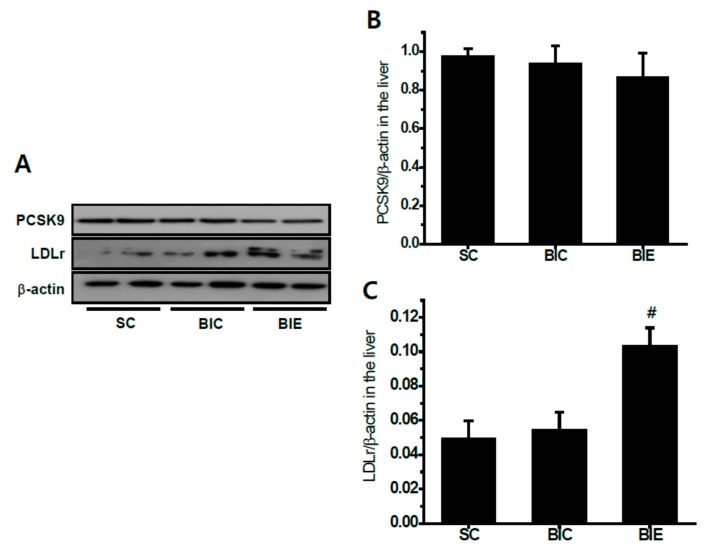
Aerobic exercise training did not affect the hepatic proprotein convertase subtilisin/kexin type 9 (PCSK9) expression but increased the low-density lipoprotein (LDL) receptor (LDLr) expression in the livers of balloon-induced rats with high-fat diets. Protein expression (**A**), and the densitometry analyses of PCSK9 (**B**) and LDLr (**C**). SC (*n* = 6), sham control; BIC (*n* = 6), balloon-injured control; BIE (*n* = 6), balloon-injured exercise. Data showed a mean ±S.E. ^#^ *p*< 0.05 vs. BIC.

We measured the hepatic PCSK9 and LDLr in the livers of rats (Figure 3). Aerobic exercise training did not significantly affect the hepatic PCSK9 protein expression. However, the LDLr expression in the liver was significantly increased by aerobic exercise compared with the BIC group (*p* < 0.05, 0.10 ± 0.02 vs. 0.05 ± 0.01). 

### 3.4. Aerobic Exercise Suppressed Expression of PCSK9, LOX-1 and VCAM-1 in CCA

**Figure 4:**
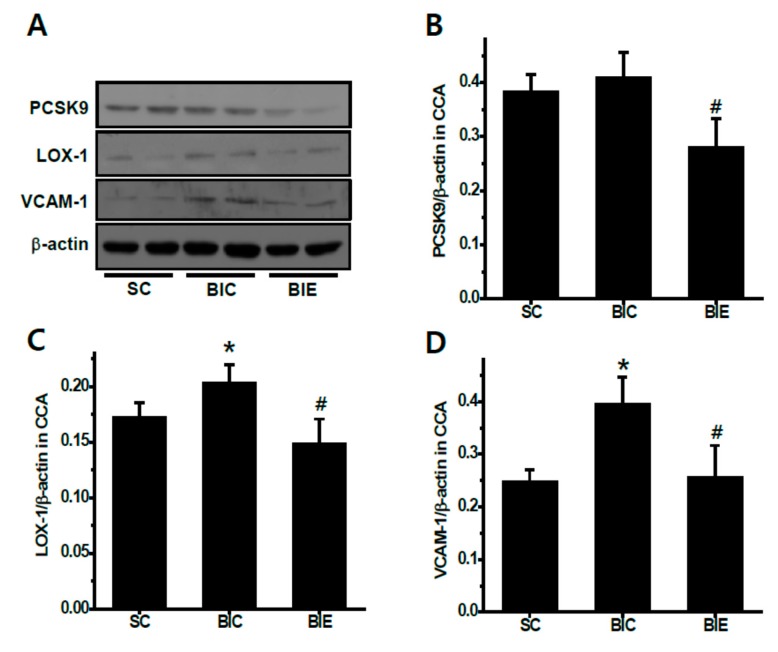
Aerobic exercise training reduced the expression of PCSK9, LOX-1 and VCAM-1 in the balloon-injured common carotid arteries (CCA) of the balloon-induced rats with high-fat diets. Protein expression (**A**), and the densitometry analyses of PCSK9 (**B**), LOX-1 (**C**) and VCAM-1 (**D**). SC (*n* = 5), sham control; BIC (*n* = 5), balloon-injured control; BIE (*n* = 5), balloon-injured exercise. Data showed a mean ±S.E. * *p* <0.05 vs. BIC; ^#^ *p* < 0.05 vs. BIE.

We investigated the expression of PCSK9, LOX-1 and VCAM-1 in the balloon-induced common carotid arteries of rats with a high-fat diet (Figure 4). Aerobic exercise training significantly inhibited the PCSK9 expression in the CCA compared to the SC and BIC group (*p* < 0.05, 0.38 ± 0.03 and 0.41 ± 0.04 vs. 0.28 ± 0.05). LOX-1 expression in the CCA was significantly increased in the BIC group compared to the SC (*p* < 0.05, 0.17 ± 0.01 vs. 0.20 ± 0.01). However, aerobic exercise training significantly recovered the increase of balloon-induced LOX-1 expression in the CCA compared to the BIC group (*p* < 0.05, 0.20 ± 0.01 vs. 0.15 ± 0.02). VCAM-1 expression in the CCA was significantly increased in the BIC compared to the SC (*p* < 0.05, 0.25 ± 0.02 vs. 0.39 ± 0.05). However, aerobic exercise training significantly recovered the increase of balloon-induced LOX-1 expression in the CCA compared to the BIC group (*p* < 0.05, 0.39 ± 0.05 vs. 0.26 ± 0.06).

## 4. Discussion

High-fat diets and physical inactivity are associated with obesity and lead to various cardiovascular system events, such as atherosclerosis. Physical activity as a therapeutic strategy to overcome obesity can effectively reduce body weight [30]. In our study, aerobic exercise training significantly inhibited weight gain in high-fat-diet rats.

Regular exercise suppresses atherosclerotic lesions in vascular walls and directly inhibits the neointimal formation as well [19,31]. Li et al. recently reported that exercise training can inhibit balloon-induced neointimal formation via overcoming endothelial dysfunction [18]. In this study, eight weeks of aerobic exercise training significantly suppressed the neointimal formation in the balloon-induced common carotid arteries of high-fat-diet rats. These findings provide direct evidence that aerobic exercise training can effectively inhibit atherosclerosis.

PCSK9 is a newly identified protein that shows potential in the treatment for dyslipidemia and atherosclerosis [32,33]. In fact, PCSK9 is mainly expressed in the liver and is secreted into circulation, and binds to LDLr on the hepatic surface, resulting in increased serum LDL-C concentrations. Experimental studies have indicated that the overexpression of PCSK9 increases plasma LDL-C level [33], but the inhibition of PCSK9 reduces LDL-C in serum [12,34].

One of the non-pharmacological strategies for the treatment of hypercholesterolemia is exercise training. Studies have recently shown controversial results that aerobic exercise training reduced the PCSK9 mRNA expression in the liver [23] of high-fat-diet obese animals. On the other hand, aerobic exercise training increased the PCSK9 mRNA expression in the liver [24] and intestines of ovariectomized rats. In the present study, exercise training did not affect the hepatic PCSK9. However, it has been consistently reported that aerobic exercise training increases the mRNA and protein expression of LDLr in the liver [23,35] and intestines [26].

In humans, LDL particles are the main carriers of cholesterol to the peripheral tissues, where they are internalized via the LDLr, a crucial mediator of plasma LDL concentrations [36]. Epidemiological evidence has consistently shown that increasing LDL concentrations were associated with the risk of vascular diseases [37]. Genetic studies have reported that early exposure to excessive LDL-C resulted in LDLr mutation and atherosclerotic lesion [38]. Exercise was shown to increase LDLr transcription in animal models [39,40]. Our results indicated that aerobic exercise significantly upregulated the hepatic LDLr protein level, which was consistent with the results of previous studies. However, considering that the main function of the hepatic LDLr is the removal of LDL-C in serum, our data showed that it effectively reduced hepatic LDLr but failed to show the positive effect of exercise training on hepatic PCSK9. It is possible that the effect of exercise training on the management of the LDL-C metabolism may take place more at the intestinal [24,25] and vasculature levels [16] than at the liver tissue, but the experimental evidence at the molecular level is unclear.

LDL-C was converted to ox-LDL by obesity-induced oxidative stress [5]. Ox-LDL was recognized by macrophages, which cause the formation of foam cells and lead to atherosclerotic plaque [41]. In addition, ox-LDL induces the apoptosis of ECs via LOX-1, whereas LOX-1 mRNA antisense inhibits ox-LDL in ECs [41]. Riahi et al. reported that chronic aerobic exercise could downregulate LOX-1 in the hearts of rats fed with a high-fat diet [42]. However, another study showed that the cessation of voluntary running significantly recovered LOX-1 in the vascular tissues [22]. A recent report indicated that LOX-1 expression was reduced in PCSK9 knockout animal models and that the overexpression of PCSK9 was reduced in LOX-1 knockout animal models [43,44,45]. These studies suggest crosstalk between PCSK9 and LOX-1 in the vascular tissues. Both LOX-1 and PCSK9 are also involved in inflammation-induced VCAM-1 [45]. VCAM-1 is expressed in endothelial cells (ECs) and smooth muscle cells (SMCs), and induces atherosclerosis [46]. Exercise training is widely known to decrease VCAM-1 expression in the vasculature [47,48,49].

Chronic endothelial injury causes atherosclerosis, but regular exercise training induces the fast recovery of damaged endothelial cells and inhibits the migration of smooth cells which is protective against balloon-induced atherosclerosis [17,19,31]. However, the role of exercise on PCSK9, LOX-1 and VCAM-1 in atherosclerosis has not been reported. Thus, we verified whether PCSK9 expression changed in atherosclerotic regions in response to exercise. Interestingly, our findings showed that aerobic exercise training significantly attenuated the expression levels of PCSK9, LOX-1 and VCAM-1 in balloon-induced sites of high-fat-diet rats.

Recently, Ding et al. suggested the possibility that an inflammatory stimulus activates the endothelial cell and smooth muscle cell to secret PCSK9, wherein induced LOX-1 activation and ox-LDL is up took, leading to atherosclerosis [43]. This study has shown a positive feedback between PCSK9 and the LOX-1 axis in the intra-arterial wall. Furthermore, it was reported that hemodynamic high shear stress reduced the expression of PCSK9 and LOX-1, but low shear stress increased the PCSK9 expression in human endothelial and smooth cells [45]. Importantly, both the PCSK9 and VCAM-1 expression are greater in aortic arch branch point and aorta–iliac bifurcation regions than in the thoracic aorta. To our knowledge, the carotid arteries used in the present study were also in bifurcation region and in low shear stress regions. Since lamina flow and high shear stress have atheroprotective effects on vascular endothelial and smooth muscle cells [45,50], it is possible that exercise-induced high shear stress may contribute to reduce the expression of PCSK9, LOX-1 and VCAM-1 in the atherosclerotic region.

Although circulating PCSK9 and LDL-C levels were not measured in the present study, our data showed at least the role of exercise training as a therapeutic option to suppress PCSK9 activation in atherosclerosis, but the evidence that it does affect PCSK9 in an isolated EC or SMC in the atherosclerotic region is lacking.

## 5. Conclusions

In conclusion, our results suggest that aerobic exercise training may increase LDLr expression in the liver and inhibits neointimal formation via the reduction of PCSK9, LOX-1 and VCAM-1 in atherosclerotic regions in the high-fat-diet-induced rat models.
